# Fish Farms at Sea: The Ground Truth from Google Earth

**DOI:** 10.1371/journal.pone.0030546

**Published:** 2012-02-08

**Authors:** Pablo Trujillo, Chiara Piroddi, Jennifer Jacquet

**Affiliations:** Fisheries Centre, University of British Columbia, Vancouver, British Columbia, Canada; University of British Columbia, Canada

## Abstract

In the face of global overfishing of wild-caught seafood, ocean fish farming has augmented the supply of fresh fish to western markets and become one of the fastest growing global industries. Accurate reporting of quantities of wild-caught fish has been problematic and we questioned whether similar discrepancies in data exist in statistics for farmed fish production. In the Mediterranean Sea, ocean fish farming is prevalent and stationary cages can be seen off the coasts of 16 countries using satellite imagery available through Google Earth. Using this tool, we demonstrate here that a few trained scientists now have the capacity to ground truth farmed fish production data reported by the Mediterranean countries. With Google Earth, we could examine 91% of the Mediterranean coast and count 248 tuna cages (circular cages >40 m diameter) and 20,976 other fish cages within 10 km offshore, the majority of which were off Greece (49%) and Turkey (31%). Combining satellite imagery with assumptions about cage volume, fish density, harvest rates, and seasonal capacity, we make a conservative approximation of ocean-farmed finfish production for 16 Mediterranean countries. Our overall estimate of 225,736 t of farmed finfish (not including tuna) in the Mediterranean Sea in 2006 is only slightly more than the United Nations Food and Agriculture Organization reports. The results demonstrate the reliability of recent FAO farmed fish production statistics for the Mediterranean as well as the promise of Google Earth to collect and ground truth data.

## Introduction

Seafood data can be fishy. When biological models could not explain the high fish catches reported off China's coast, scientists realized Chinese officials were intentionally inflating the capture fisheries statistics [1]. More commonly, searching the gray literature can reveal that countries underreport their fish catches to the United Nations Food and Agriculture Organization (FAO) due to lack of oversight and/or high levels of subsistence fishing [2]. Discrepancies between reported and actual use for any resource can contribute to poor science and policy, and can justify scrutinizing reports and re-approximating data from the ground up. For the first time, we use Google Earth to estimate the farmed fish production in the Mediterranean Sea, which we compare to data on farmed fish production provided by each Mediterranean country provided to the FAO, the organization mandated to collect data for capture fisheries and farmed fish production, in 2006 (excluding Serbia Montenegro, which did not report marine finfish aquaculture for that year).

Due to extensive and expanding overfishing of wild-caught seafood [3], [4], ocean fish farming has grown to augment the supply of fresh fish to western markets. Indeed, it is one of the fastest growing global industries [5]. The accuracy of available data for farmed fish is important to gauge the magnitude and growth of this industry, its role in feeding global seafood demand, and also for determining the industry's impact on small pelagic fish because farmed fish currently require large quantities of wild fish for fishmeal and oil [6]. Ocean fish farming began in the Mediterranean in the early 1980s and is now widespread. Stationary cages speckle the coasts of 16 Mediterranean countries and are visible from satellite imagery available through Google Earth. Google Earth has shown great promise scientifically, for instance in geo-referencing and mapping [7], teaching geological science [8], identifying new sites of archeological importance (*e.g.*, [9]), and in examining predator-prey interactions on coral reefs [10]. We wanted to explore Google Earth's potential to ground truth farmed fish production data.

## Results and Discussion

Of the entire Mediterranean coast, Google Earth satellite images were available for 91% of the Mediterranean shores. We identified and counted 248 tuna cages (circular cages >40 m diameter [11] and 20,976 other fish cages within 10 km offshore, the majority of which were off Greece (49%) and Turkey (31%). Around 80% of cages are located within 100 km of shore, and details for number of cages per country and the nearest and furthest cages from shore are provided in Table 1.

**Table 1:** Number of cages, closest and furthest cage to shore, average area per cage, and the various assumptions used to estimate finfish production for each Mediterranean country.

Country	No. of cages	Cage closest to shore (m)	Cage furthest from shore (m)	Average cage/pen area (m^2^)	Estimated average fish density (kg/m^3^)	Ratio of production Sea bream to Sea bass	Estimates of harvest cycle Sea bream/Sea bass (months)	Production estimate assuming 50% of cages in production (mt)	Production estimate assuming 100% of cages in production (mt)	Production estimate assuming 75% of cages in production (mt)	Reported production to FAO for 2006 (mt)
Greece	10,422	25	316	130	15 [11], [16], [17]	1.3∶1 [18]	13/15 [11], [19]	69213	138426	103819	79534 [18]
Turkey	6,512	12	393	205	15 [11], [20], [21]	0.7∶1 [18]	12/14 [11], [19], [21]	54558	109115	81836	69071 [18]
France	1,213	67	988	55	12 [11]	0.5∶1 [18]	16/18 [11], [19]	1339	2678	2008	5130 [18]
Italy	912	20	2300	217	12 [11], [22]	0.9∶1 [18]	15/18 [11], [19]	4691	9383	7037	12740 [18]
Croatia	751	28	440	240	12 [11], [23]	0.4∶1 [18]	16/18 [11], [19]	2912	5133	3850	3422 [18]
Spain	573	240	7023	700	15 [11], [14], [24]	2.2∶1 [18]	16/20 [11], [19], [25]	14125	28251	21188	21350 [18]
Cyprus	129	450	1326	405	15 [11], [26]	3.2∶1 [18]	13/15 [11], [19]	1915	3831	2873	2470 [18]
Tunisia	92	500	560	298	12 [11]	1.3∶1 [18]	13/15 [11], [19]	697	1395	1046	1140 [18]
Malta	52	115	460	142	12 [11]	5.9∶1 [18]	13/15 [11], [19]	373	746	559	1096 [18]
Bosnia-Herzegovina	52	30	47	94	12 [11]	1.2∶1 [18]	16/18 [11], [19]	126	253	190	183 [18]
Slovenia	38	750	950	60	12 [11]	0∶1 [18]	16/18 [11], [19]	48	96	72	30 [18]
Albania	148	25	230	72	12 [11]	1∶0 [18]	16/18 [11], [19]	305	611	458	370 [18]
Libya	21	500	580	193	12 [11]	0.4∶1 [18]	13/15 [11], [19]	183	365	274	230 [18]
Serbia-Montenegro	28	110	135	29	12 [11]	1∶1 [18]	16/18 [11], [19]	45	89	67	0 [18]
Israel	18	20	30	254	12 [11]	73∶1 [18]	13/15 [11], [19]	262	525	394	2725 [18]
Morocco	15	480	620	107	12 [11]	1.3∶1 [18]	16/18 [11], [19]	43	87	65	51 [18]
TOTAL	20,976	–	–	–	–	–	–	150,835	300,984	225,736	199,542 [18]

We provide three different estimates of finfish production based on different assumptions about the percentage of cages (50, 75, 100) that are fully operational. The final column provides the reported finfish production to FAO in 2006 by country.

**Figure 1:**
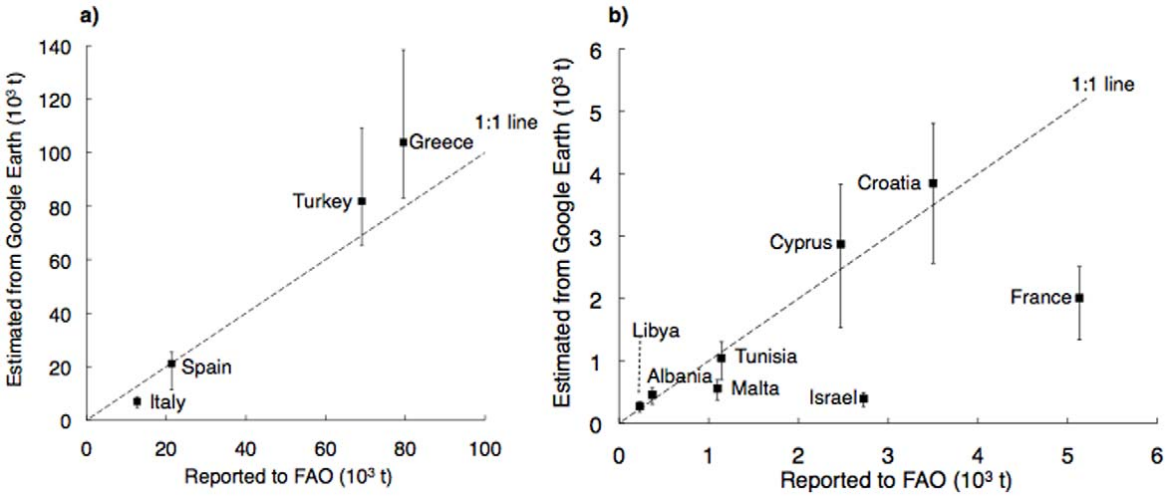
Average production estimates (black boxes) and ranges (bars) for ocean cage farmed finfish production (assuming 75% of cages in production and not including tuna) compared with official finfish data reported to FAO. Fig. (a) reports estimates for the countries with the largest finfish production and (b) for the countries with smaller production. Estimates for France and Israel are much lower than FAO data probably due to low cage counts as a result of inadequate satellite coverage of both countries' coasts.

We excluded tuna ranches from further analysis, because tuna production highly fluctuates due to reliance on captured wild juvenile fish [12]. After analyzing approximately 25 shellfish farm sites, we excluded seaweed and shellfish from our analysis due to challenges in assuming production rates and area coverage. Combining satellite imagery with assumptions about cage volume, fish density, harvest rates, and capacity of production, we estimated 225,736 tonnes (t) of farmed finfish (not including tuna) were produced in the Mediterranean Sea in 2006. Estimated ocean farmed fish production (excluding tuna) above 25 t is available in Fig. 1 (we excluded those countries with a production below 250 t: Bosnia-Herzegovina 190 t, Slovenia 72 t, Serbia-Montenegro 67 t, Morocco 65 t).

Greece led farmed ocean finfish production with 103,819 t in 2006 followed by Turkey, Spain, and Italy. Our estimates for Greece were ∼30% higher than the values reported that year to FAO, ∼18% higher than what Turkey reports, ∼16% higher than Cyprus and ∼10% higher than Croatia's official FAO values. Conversely, Italy, France, Malta and Israel reported higher values to FAO, which could be due to the confusion of imports as actual production by the country (*e.g.*, Italy appears to label Greek imports as Italian production (observed by authors PT and CP) or due to the lack of imagery for parts of the coast. For reasons unknown to us, images for ∼40% of France's coast – mostly around Provence and Corsica, the major producing regions in the French Mediterranean coast [11]– were not available. In the satellite images of Israel's coast, low resolution and outdated imagery prevented the detection of the majority of the cages.

Contrary to many cases of capture fisheries [2], our overall estimate of 225,736 is close to the 2006 FAO reported figure of 199,542 t of farmed ocean finfish, not including tuna, for the Mediterranean. For the 16 countries examined here, the difference between the FAO data and our estimates is not significant (2-sample *t*-test, *n*_1_ = 16, *n*_2_ = 16, *t* = 0.16, *P* = 0.87; test is 2-tailed and the country is the statistical unit). This is not particularly surprising because ocean cage farmed fish are a comparatively new, high value, capital intense product destined for market sale (while capture fisheries destined for subsistence consumption are often overlooked). However, our methods required several assumptions and might have underestimated production due to the lack of photographs and/or resolution, especially along the coasts of France and Israel.

Despite some missing imagery, the Mediterranean Sea was an ideal location to ground truth farmed fish production due to the prevalence of fish farms and the high fraction of coast covered by Google Earth relative to other parts of the world. In addition, using Google Earth we could obtain higher resolution data (i.e., number and location of cages) compared to those available through FAO.

Although we had to make assumptions about species, production, and seasonal capacity, our estimates do not rely on industry reports, one traditional source of information. The results here demonstrate the promise of the new and untraditional tool of Google Earth to collect and ground truth data. We show that a few people are now capable of estimating farmed fish production for 16 countries if they have the training, the patience to meticulously examine the coast, and the Internet.

## Materials and Methods

We scanned the coasts (10 km offshore or less) of each country at varying resolutions multiple times and assigned a place mark to each aggregation of fish cages. Using the Google Earth time slider tool, we were able to assign also a temporal scale to each satellite picture (available from 2002–2010). We chose to estimate production for 2006 because this year yielded the highest number of satellite images of the coast.

We used the Google's Earth ruler tool (calibrated using images of tennis courts) to obtain fish cage diameters or widths and then calculate cage area (168 m^2^ median; 113 m^2^ mode). For information on average cage width in each country, see Table 1. Based on scientific and industry reports on cage dimensions [13], [14] as well as bathymetric constraints, we inferred the depth of cages (*e.g.*, 7.6 m average in Greece; 16.4 m average in Spain), which we used to calculate volume (2097 m^3^ median; 904 m^3^ mode). We also searched the literature to estimate species composition and ratio of production in each country (almost entirely Gilthead seabream, *Sparus aurata*, and European seabass, *Dicentrarchus labrax*; note that the only time we used FAO statistics was to calculate the ratio of ocean farmed seabream to seabass), average fish density, and estimates of harvest cycle (Table 1). (Fish raised in countries with colder winter temperatures would have slower growth rates and therefore later harvest times. Based on sea surface temperatures (<16°C), we determined that Spain, France, northern and central Italy, Croatia, Albania, Slovenia, and Bosnia-Herzegovina require overwintering of the fish and therefore have delayed harvest times). Just as farmers let fields lie fallow, not all fish cages are operational at all times. Based on data available in the images (*e.g.*, oily runoff around cages or nets covering cages indicate active farms [15]) we report estimates in the main text for the assumption that 75% of cages were in production. In Table 1, we additionally report the total productions, assuming that 50% and 100% of cages are in production, both of which are less likely scenarios. Our assumption that 75% of cages are producing fish takes into account that not all cages are active or at optimal capacity at all times (they might be fallow or being used for raising juveniles), and therefore provides a conservative estimate of production.

Official data on farmed seafood production are available through the database FishStat (see http://www.fishstat.org) as part of the mandate of the FAO, whose constitution requires the collection, analysis, interpretation, and dissemination of information related to nutrition, food, and agriculture. Since 1950, FAO has compiled, from every country, reported farmed fish production and related data broken down into weight by taxa. We used the FAO reported finfish production (minus tuna) in 2006 for the countries described here as the basis of our comparison.
