# Correction: Fish Farms at Sea: The Ground Truth from Google Earth

**DOI:** 10.1371/journal.pone.0134745

**Published:** 2015-07-29

**Authors:** The PLOS ONE Staff

## Notice of Republication

This article was republished on June 23, 2015, to replace copyrighted or confidential material. Please download this article again to view the correct version.
